# Global, regional, and national burdens of PUD in women of reproductive age from 1992 to 2021: a trend analysis based on the global burden of disease study 2021

**DOI:** 10.3389/fgwh.2025.1529549

**Published:** 2025-04-01

**Authors:** Xiaofeng Wang, Song Yang, Shanzhi Zhao, Zhitao Yang, Enqiang Mao, Erzhen Chen, Ying Chen

**Affiliations:** ^1^ Department of Emergency, Ruijin Hospital, Shanghai Jiao Tong University School of Medicine, Shanghai, China; ^2^ Department of Emergency and Critical Care Medicine, Ruijin Hospital Wuxi Branch, Shanghai Jiao Tong University School of Medicine, Wuxi, China; ^3^ Shanghai Jiao Tong University Medical School Affiliated Ruijin Hospital, Shanghai Institute of Aviation Medicine, Shanghai, China

**Keywords:** peptic ulcer disease, women of childbearing age, predictive modeling, global burden of disease, ASIR

## Abstract

**Background:**

Peptic ulcer disease (PUD) constitutes a significant global health concern, particularly in women of childbearing age (WCBA), who face elevated risks of severe pregnancy-associated complications. This investigation aimed to map the temporal dynamics and forecast the future incidence of PUD in this demographic to inform targeted prevention and control initiatives.

**Methods:**

This analysis drew on the Global Burden of Diseases, Injuries, and Risk Factors Study (GBD) 2021, extracting data on PUD incidence and mortality across seven age groups (15–49 years) in WCBA. Age-standardized incidence and mortality rates were calculated using the direct method of age standardization. Temporal trends from 1992 to 2021 were analyzed using joinpoint regression. The study further employed age-period-cohort analysis to discriminate the effects of these variables on incidence and mortality, and frontier analysis to evaluate potential reductions in burden by country based on developmental status. Nordpred modeling was used to project epidemiological trends up to 2044.

**Results:**

In 2021, the global age-standardized incidence rates (ASIR) and death rates (ASDR) for PUD among WCBA were 24.18 per 100,000 (95% CI: 14.72–36.38) and 0.54 per 100,000 (95% CI: 0.42–0.66), respectively. The highest incidence rates were observed in Oceania, while the greatest mortality rates were recorded in South Asia. Over the period from 1992 to 2021, global age-standardized mortality rates showed a significant decline. Conversely, after an initial drop, age-standardized incidence rates began to rise, with considerable regional and country-specific variation. This increase was particularly marked in regions with high Socio-demographic Index (SDI). Frontier analyses indicate that countries or regions in the middle SDI quintiles possess significant untapped potential to enhance both access to and quality of healthcare. Despite predictions of declining age-standardized incidence and mortality rates, total case numbers are expected to continue rising modestly through 2044.

**Conclusions:**

The study underscores substantial global disparities in PUD trends in WCBA, with increasing case numbers and regional inequalities. The findings highlight the need for focused attention on high SDI regions and older WCBA cohorts to refine disease management and prevention strategies, aiding in the mitigation of PUD's public health impact.

## Introduction

Peptic ulcer disease (PUD) arises from an imbalance between aggressive factors that damage the mucosa and the defensive mechanisms that safeguard it (1). Globally prevalent, this condition not only diminishes quality of life through chronic pain and malnutrition but also poses substantial health risks (2). The annual incidence of PUD is approximately one case per 1,000 person-years in the general population, with each individual facing a lifetime risk of 5%–10% (3). The risk factors for this disease are multifactorial. Historically, it was attributed to hyper-secretory acidic environments, stress, and dietary factors, but the understanding of PUD etiology shifted dramatically in the latter half of the 20th century with the identification of Helicobacter pylori (H. pylori) and the widespread use of non-steroidal anti-inflammatory drugs (NSAIDs) (4–6).

The clinical presentation of PUD is characteristically nonspecific. Typical symptoms of gastric ulcers include postprandial abdominal pain, nausea, and weight loss, while duodenal ulcers often manifest as hunger pangs or nocturnal pain (1). While many cases of PUD are self-limiting, complications such as bleeding or perforation can be life-threatening and are associated with stable yet concerning case fatality rates (7). Definitive diagnosis of PUD requires invasive upper endoscopy, a procedure often avoided by gastroenterologists due to technical difficulties and concerns regarding fetal and maternal safety. Consequently, PUD may be underdiagnosed during pregnancy. Additionally, hormonal fluctuations and physiological adaptations during pregnancy may worsen preexisting ulcers or impede healing. Untreated PUD in WCBA is associated with significantly higher rates of hemorrhagic and anemic complications, which are linked to adverse outcomes, including preterm birth, intrauterine growth restriction, and maternal mortality (8). Accordingly, PUD is particularly significant for women of childbearing age (WCBA), who experience heightened risks of severe pregnancy-related complications that contribute to substantial maternal morbidity and mortality. Alarmingly, there exists a concerning global shortfall in research addressing the incidence of PUD within this critical demographic, indicating that the unique vulnerabilities of WCBA remain insufficiently explored.

The economic impact of PUD is profound, including direct healthcare expenditures—such as prolonged pharmacological treatment and hospitalization—and indirect societal costs, such as absenteeism and diminished productivity resulting from chronic pain or fatigue (9). PUD exhibits the greatest health inequality among nonmalignant upper gastrointestinal diseases, though its disparity has markedly decreased from 1990 to 2019 (10). Beyond the direct costs associated with medical care and hospitalization, the disease also incurs significant indirect costs, including lost labor and diminished productivity (11). Recent studies, however, indicate that middle socioeconomic regions might also bear a disproportionate burden of the disease, and the burden in certain female age groups has begun to exceed that of their male counterparts, challenging previous assumptions about its demographic and geographic distribution (12, 13).

Drawing from the Global Burden of Diseases, Injuries, and Risk Factors Study (GBD) 2021, which evaluated the global impact of 371 diseases and 88 risk factors across 204 countries (14). This study leverages the comprehensive dataset to analyze the epidemiological trends of PUD in WCBA from 1992 through 2021. By analyzing age, period, and cohort effects on the disease burden and forecasting future incidence and mortality, this research aims to establish a robust empirical foundation for developing targeted and effective prevention and intervention strategies.

## Materials and methods

### Study population and data collection

The PUD data among WCBA analyzed in this study were derived from the 2021 GBD Study using the GBD Outcomes Tool, which provides a comprehensive scientific assessment of published, publicly available, and contributory incidence, prevalence, and mortality data for 369 diseases, injuries, and impairments, as well as 88 risk factors across 21 GBD regions and 204 countries and territories (14). As defined by the World Health Organization (WHO), WCBA refers to individuals aged 15–49 years (15). To summarize the age distribution of the burden of PUD in WCBA, patients were categorized into 7 groups: 15–19 years, 20–24 years, 25–29 years, 30–34 years, 35–39 years, 40–44 years, 45–49 years. The burden of PUD among WCBA was quantified over time, by region, and by age using the direct method; specifically, it is assumed that the incidence distribution follows a weighted sum of independent Poisson random variables, employing age-standardized incidence rates (ASIR) and age-standardized death rates (ASDR)s (16).

The Socio-demographic Index (SDI) is a comprehensive indicator representing the development status of a country or region, which is based on the overall fertility rate among females under the age of 25 years, the average education level of individuals aged over 15 years, and the country's lag-distributed income per capita. The SDI takes values from 0 to 1, where 0 represents the lowest socioeconomic development. In contrast, 1 indicates the highest socioeconomic development. Based on their SDI values, the 204 countries and regions in GBD 2021 were categorized into five groups: low, low-middle, middle, high-middle, and high SDI (17).

### Temporal trend in PUD burden

We employed Joinpoint regression model as a statistical tool to assess trends in disease burden over time (18). The Joinpoint Regression Program (version 4.9.1.0) was utilized to evaluate trends in PUD among WCBA from 1990 to 2021. The model facilitates the identification of significant changes in trends by calculating annual percentage changes (APCs) and their corresponding 95% confidence intervals (CIs) (19). Additionally, an average annual percentage change (AAPC) was calculated to assess trends, with a weight corresponding to the length of each period within a specified time interval. A trend is considered constant when the value of zero falls within the 95% CI of the AAPC. When both limits of the 95% confidence interval are positive, the trend is classified as upward; conversely, when both limits are negative, the trend is classified as downward (20).

### Age-period-cohort trends of PUD prevalence

The age-period-cohort (APC) model has been recognized and employed in assessing the impact of age, period, and cohort effects on health outcomes (21–23). Given the perfectly linear relationship among age, period, and cohort, estimating their independent effects is statistically infeasible; therefore, we circumvent this issue by generating estimable APC parameters and functions that impose no arbitrary constraints on the model parameters. Net drift represents the overall log-linear trend by period and birth cohort, indicating the overall annual percentage change in the expected age-standardized rate (ASR) over time (24). The Wald *χ*^2^ test was performed to access the significance of trends in annual percentage change. Longitudinal age curve, which describe the age-specific and cohort-specific rates adjusted for period bias, are generally regarded as superior to a cross-sectional age curve for accessing age effects. The relative risk (RR) of a cohort (or period) represents the relative risk associated with that cohort or period, compared to a reference cohort or period, adjusted for age and nonlinear period or cohort effects. An RR value greater than 1 indicates that the factor elevates the risk of PUD incidence or mortality. Conversely, when the RR value is less than 1, it indicates that the factor diminishes the risk of PUD morbidity or mortality (25).

In a typical APC model, age intervals are required to correspond to period intervals. Therefore, seven distinct age groups, each spanning 5 years (15–19, 20–24, 25–29, 30–34, 35–39, 40–44, and 45–49 years), were selected for further analysis (21). Correspondingly, the period from 1992 to 2021 was divided into six 5-year periods (1992–1996, 1997–2001, 2002–2006, 2007–2011, 2012–2016, 2017–2021). Additionally, twelve consecutive birth cohort groups, each spanning 5 years, were also defined (1947–1951, 1952–1956, 1957–1961, 1962–1966, 1967–1971, 1972–1976, 1977–1981, 1982–1986, 1987–1991, 1992–1996, 1997–2001, 2002–2006).

### Frontier analysis

To evaluate the efficiency and performance of different regions in managing the burden of PUD in WCBA, we employed a frontier analysis (26). To derive a non-linear frontier, we employed a non-parametric data envelopment analysis and referenced detailed descriptions from previous studies. This frontier represents the lowest achievable burden determined, contingent upon the level of development. The distance between a country's observed ASIR and its frontier-defined as the effective difference-reflects the unrealized health gains in relation to the country's current level of development.

### Prediction analysis

To estimate the burden of PUD in WCBA over the next 23 years, the incidence, mortality, ASIR, and ASDR of PUD among WCBA were projected using Nordpred package (27). This package considers shifting demographics and rates, which demonstrates its effectiveness in predicting the future disease incidence trends and mortality trends. First, download demographic data for PUD in WCBA from 1992 to 2021 from the GBD 2021 database, and access the predicted demographic data available at the following link (https://ghdx.healthdata.org/data-type/estimate). Subsequently, use the Nordpred package to project the future burden of PUD in WCBA.

R (version 4.3.2, R core team) software was used for the statistical procedures. The relationships between ASIR, ASDR, and SDI were assessed using Pearson's correlation analysis. A *p*-value of <0.05 was considered statistically significant.

## Results

### Trends in the morbidity and mortality of PUD in WCBA at the global, regional, and national levels

Globally, the estimated global ASIR and ASDR of PUD in WCBA were 24.18 [95% confidence interval (CI): 14.72–36.38] and 0.54 (95% CI: 0.42–0.66) in 2021, respectively (Tables 1, 2). From 1992 to 2021, the global ASIR estimate for PUD among WCBA showed a significant downward trend (AAPC = −1.18%, 95% CI: −1.25% to −1.10%; *P* < 0.001), with the largest change occurring from 2005 to 2014 (APC = −2.18, *P* < 0.001) (Supplementary Table S3). At the same time, the global ASDR estimated for PUD among WCBA demonstrated a significant decrease (AAPC = −3.15%, 95% CI: −3.31% to −3.00%; *P* < 0.001), with the most substantial changes occurring from 2000 to 2015 (APC = −3.95, *P* < 0.001) (Supplementary Table S3).

**Table 1:** The incident cases and ASIR of PUD in women of reproductive age in 1992 and 2021 at global and regional levels.

Location	Incidence number in 1992	Incidence number in 2021	ASIR in 1992	ASIR in 2021
Global	4,60,921.88 (2,78,133.77, 7,00,658.38)	4,73,628.97 (2,88,656.15, 7,12,328.69)	34.21 (52, 20.7)	24.18 (14.72, 36.38)
SDI quintiles				
High SDI	48,762.92 (27,655.38, 77,655.8)	57,490.45 (33,648.14, 89,247.98)	20.52 (11.62, 32.67)	21.87 (12.75, 33.96)
High-middle SDI	62,719.11 (36,022.01, 98,980.48)	56,885.12 (33,314.53, 88,707.65)	22.53 (13.01, 35.49)	16.91 (9.8, 26.45)
Middle SDI	1,42,068.68 (84,295.59, 2,17,673.67)	1,23,705.89 (73,599.38, 1,88,901.5)	32.58 (19.43, 49.86)	19.6 (11.62, 29.95)
Low-middle SDI	1,53,149.62 (95,042.79, 2,27,509.92)	1,53,915.51 (97,025.8, 2,25,813.3)	54.74 (34.04, 81.15)	30.57 (19.32, 44.81)
Low SDI	53,903.73 (32,811.82, 80,781.86)	81,273.85 (50,006.37, 1,20,304.27)	45.71 (27.85, 68.6)	29.01 (17.98, 42.73)
GBD regions				
Andean Latin America	1,765.54 (1,037.68, 2,691.75)	2,037.12 (1,215.03, 3,081.38)	18.91 (11.29, 28.58)	11.69 (6.98, 17.68)
Australasia	608.4 (330.75, 1,001.27)	749.67 (414.01, 1,216.72)	10.65 (5.78, 17.53)	9.6 (5.26, 15.63)
Caribbean	1,477.24 (841.62, 2,315.91)	1,763.66 (1,037.55, 2,710.96)	16.04 (9.24, 25)	14.6 (8.56, 22.49)
Central Asia	3,571.67 (2,087.47, 5,464.27)	5,247.91 (3,185.65, 7,875.97)	22.19 (13.16, 33.67)	21.25 (12.87, 31.92)
Central Europe	7,669.86 (4,469.47, 11,913.23)	6,715.3 (4,121.15, 10,016.87)	23.89 (13.88, 37.15)	23.24 (14.09, 34.79)
Central Latin America	3,545.86 (2,098.05, 5,460.74)	3,595.23 (2,127.52, 5,561.85)	9.23 (5.58, 14.09)	5.19 (3.06, 8.04)
Central Sub-Saharan Africa	3,292.98 (1,954.52, 5,003)	8,121.6 (4,892.35, 12,098.83)	24.72 (14.85, 37.3)	24.29 (14.79, 35.98)
East Asia	1,02,496.22 (59,564.69, 1,60,360.76)	65,450.33 (38,519.38, 1,02,196.63)	32.16 (18.84, 50.13)	16.92 (9.84, 26.51)
Eastern Europe	9,705.26 (5,594.6, 15,203.6)	11,172.55 (6,846.65, 16,694.1)	17.07 (9.86, 26.74)	20.36 (12.28, 30.72)
Eastern Sub-Saharan Africa	12,801.12 (7,694.78, 19,211.92)	22,055.26 (13,221.36, 32,912.91)	26.23 (15.77, 39.47)	19.35 (11.6, 28.97)
High-income Asia Pacific	8,911.86 (4,973.32, 14,445.8)	7,656.95 (4,384.56, 12,348.65)	18.83 (10.47, 30.52)	17.46 (9.84, 28.28)
High-income North America	21,158.49 (12,023.11, 33,682.86)	24,063.98 (14,401.28, 36,851.99)	26.89 (15.25, 42.8)	27.46 (16.41, 42.06)
North Africa and Middle East	23,050.79 (13,321.94, 35,802.95)	41,050.43 (23,860.16, 63,950.85)	29.19 (17.09, 45.03)	25.73 (14.96, 40.06)
Oceania	820.11 (502.29, 1,225.24)	1,574.27 (967.27, 2,328.31)	52.56 (32.65, 77.95)	46.43 (28.68, 68.51)
South Asia	1,82,989.16 (1,13,556.43, 2,73,524.88)	1,65,613.19 (1,04,960.53, 2,43,391.98)	69.15 (42.95, 103.26)	33.63 (21.36, 49.4)
Southeast Asia	38,918.1 (22,673.25, 59,764.8)	45,557.11 (26,593.29, 69,908.18)	33.14 (19.46, 50.82)	24.5 (14.27, 37.6)
Southern Latin America	951.79 (519.82, 1,559.22)	1,294.86 (715.35, 2,101.41)	7.62 (4.17, 12.47)	7.14 (3.92, 11.6)
Southern Sub-Saharan Africa	3,829.93 (2,261.17, 5,897.93)	4,107.12 (2,440.65, 6,224.43)	26.24 (15.51, 40.31)	18.82 (11.19, 28.54)
Tropical Latin America	6,721.23 (3,976.27, 10,292.88)	5,735.96 (3,310.66, 9,018.63)	17.74 (10.58, 27.07)	9.09 (5.21, 14.31)
Western Europe	10,017.17 (5,602.98, 16,389.85)	9,189.65 (5,158.84, 14,785.49)	9.88 (5.5, 16.19)	8.74 (4.85, 14.13)
Western Sub-Saharan Africa	16,619.11 (9,734.95, 25,664.47)	40,876.83 (24,555.48, 61,552.66)	32.93 (19.47, 50.5)	31.54 (19.1, 47.08)

SDI, socio-demographic index; ASIR, age-standardized incidence rate.

**Table 2:** The deaths and ASDR of PUD in women of reproductive age in 1992 and 2021 at global and regional levels.

Location	Deaths in 1992	Deaths in 2021	ASDR in 1992	ASDR in 2021
Global	17,663.03 (13,879.66, 22,446.56)	10,707.46 (7,881.61, 13,787.14)	1.36 (1.13, 1.64)	0.54 (0.42, 0.66)
SDI quintiles				
High SDI	542.86 (513.96, 576.64)	247.51 (227.93, 274.73)	0.22 (0.21, 0.24)	0.09 (0.08, 0.1)
High-middle SDI	1,164.3 (983.52, 1,368.5)	689.61 (612.82, 775.88)	0.43 (0.36, 0.51)	0.19 (0.17, 0.21)
Middle SDI	4,020.03 (3,396.59, 4,798.48)	2,106.18 (1,744.34, 2,590.63)	0.98 (0.83, 1.16)	0.32 (0.26, 0.39)
Low-middle SDI	8,484.12 (6,483.7, 11,004.7)	4,714.9 (3,308.36, 6,226.77)	3.29 (2.54, 4.23)	0.97 (0.68, 1.29)
Low SDI	3,451.72 (2,501.89, 4,698.24)	2,949.26 (1,988.16, 3,919.13)	3.31 (2.43, 4.47)	1.18 (0.79, 1.57)
GBD regions				
Andean Latin America	100.58 (71.43, 139.2)	34.74 (22.78, 51.02)	1.15 (0.81, 1.59)	0.2 (0.13, 0.29)
Australasia	8.2 (6.82, 9.76)	1.82 (1.48, 2.21)	0.14 (0.12, 0.17)	0.02 (0.02, 0.03)
Caribbean	79.17 (55.3, 113.57)	62.71 (38.14, 103.09)	0.92 (0.65, 1.32)	0.51 (0.31, 0.84)
Central Asia	116.87 (104.26, 131.64)	142.08 (118.05, 171.85)	0.78 (0.69, 0.88)	0.56 (0.47, 0.68)
Central Europe	168.33 (154.65, 185.17)	104.35 (92.37, 116.7)	0.51 (0.46, 0.56)	0.32 (0.28, 0.35)
Central Latin America	323.57 (296.42, 354.94)	192.96 (157.03, 235.52)	0.87 (0.8, 0.95)	0.28 (0.23, 0.34)
Central Sub-Saharan Africa	136.22 (49.47, 247.93)	245.35 (110.34, 440.91)	1.23 (0.44, 2.25)	0.87 (0.39, 1.56)
East Asia	2,401.6 (1,809.54, 3,063.88)	539.32 (388.62, 746.45)	0.78 (0.59, 0.99)	0.14 (0.1, 0.19)
Eastern Europe	272.64 (258.82, 287.85)	383.37 (330.53, 443.59)	0.49 (0.46, 0.51)	0.62 (0.53, 0.72)
Eastern Sub-Saharan Africa	825.3 (456.62, 1,301.82)	957.13 (516.17, 1,442.53)	2.01 (1.11, 3.18)	0.94 (0.5, 1.43)
High-income Asia Pacific	67.06 (55.09, 82.93)	10.56 (8.71, 13.15)	0.14 (0.11, 0.17)	0.02 (0.02, 0.03)
High-income North America	129.99 (122.97, 137.56)	81.37 (75.33, 87.8)	0.16 (0.15, 0.17)	0.09 (0.08, 0.09)
North Africa and Middle East	557.26 (411.55, 741.82)	415.58 (284.14, 575.49)	0.78 (0.58, 1.04)	0.26 (0.18, 0.36)
Oceania	24.53 (13.59, 41.35)	29.53 (16.89, 48.08)	1.69 (0.95, 2.83)	0.9 (0.51, 1.46)
South Asia	10,148.25 (7,723.7, 13,093.33)	5,261.89 (3,440.84, 7,341.31)	4.15 (3.19, 5.31)	1.1 (0.72, 1.54)
Southeast Asia	1,254.01 (817.85, 2,027.72)	831.95 (604.78, 1,122.6)	1.1 (0.72, 1.77)	0.44 (0.32, 0.59)
Southern Latin America	26.42 (22.37, 31.24)	14.75 (12.39, 17.66)	0.21 (0.18, 0.25)	0.08 (0.07, 0.1)
Southern Sub-Saharan Africa	103.85 (72.01, 142.53)	126.9 (89.7, 171.61)	0.87 (0.6, 1.18)	0.61 (0.43, 0.82)
Tropical Latin America	177.1 (164.31, 191.35)	154.79 (140.85, 169.46)	0.49 (0.45, 0.52)	0.23 (0.21, 0.26)
Western Europe	202.64 (189.51, 216.29)	45.31 (42.46, 48.61)	0.2 (0.18, 0.21)	0.04 (0.04, 0.04)
Western Sub-Saharan Africa	549.4 (372.41, 859.73)	1,078.4 (723.59, 1,474.87)	1.33 (0.9, 2.07)	0.97 (0.66, 1.32)

SDI, socio-demographic index; ASDR, age-standardized death rate.

Across the five SDI regions, the ASDR of PUD decreased from the low SDI quintile (1.18, 95% CI: 0.79–1.57) to the high SDI quintile (0.09, 95% CI: 0.08–0.10), whereas the ASIR was the highest in the low-middle SDI quintile (30.57, 95% CI: 19.32–44.81) and lowest in the middle-high SDI quintile (16.91, 95% CI: 9.80–26.45) in 2021 (Tables 1, 2). From 1992 to 2021, the increasing trend in ASIR was only observed in high SDI regions, while decreases were noted in the remaining four regions. ASDR is experiencing a downward trend across all regions, including those with high SDI. A comparable pattern is observed in the net drift results derived from the APC model (Supplementary Table S4).

**Figure 1:**
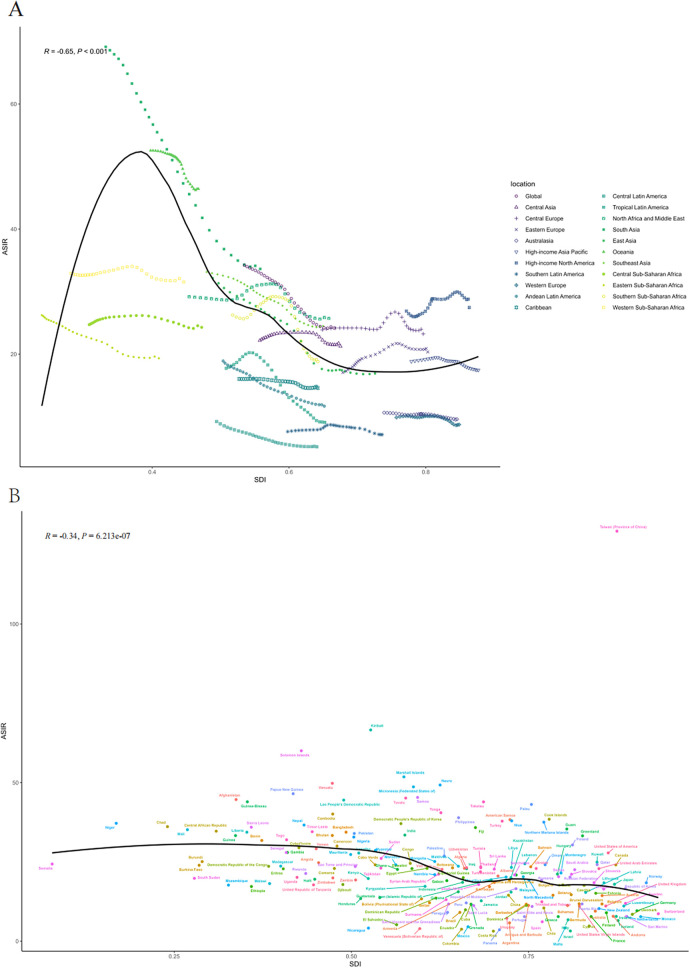
The ASIR of PUD in women of reproductive age for 21 GBD regions from 1992 to 2021 **(A)** and 204 countries and territories in 2021 **(B)** by SDI. SDI, socio-demographic index; ASIR, age-standardized incidence rate.

By geographical category, the highest estimated ASIR and ASDR of PUD in WCBA were in Oceania and South Asia, while the lowest estimated ASIR occurred in Central Latin America and ASDR occurred in Australasia and high-income Asia Pacific in 2021, respectively (Tables 1, 2). From 1992 to 2021, the most significant decrease of ASIR and ASDR occurred in the South Asia, while the increase of ASIR occurred in East Europe and High-income North America and the increase of ASDR only occurred in East Europe (Supplementary Table S3). In general, the ASIR and ASDR for PUD among WCBA exhibited a negative correlation with SDI levels (Figure 1A, Supplementary Figure S2A).

**Figure 2:**
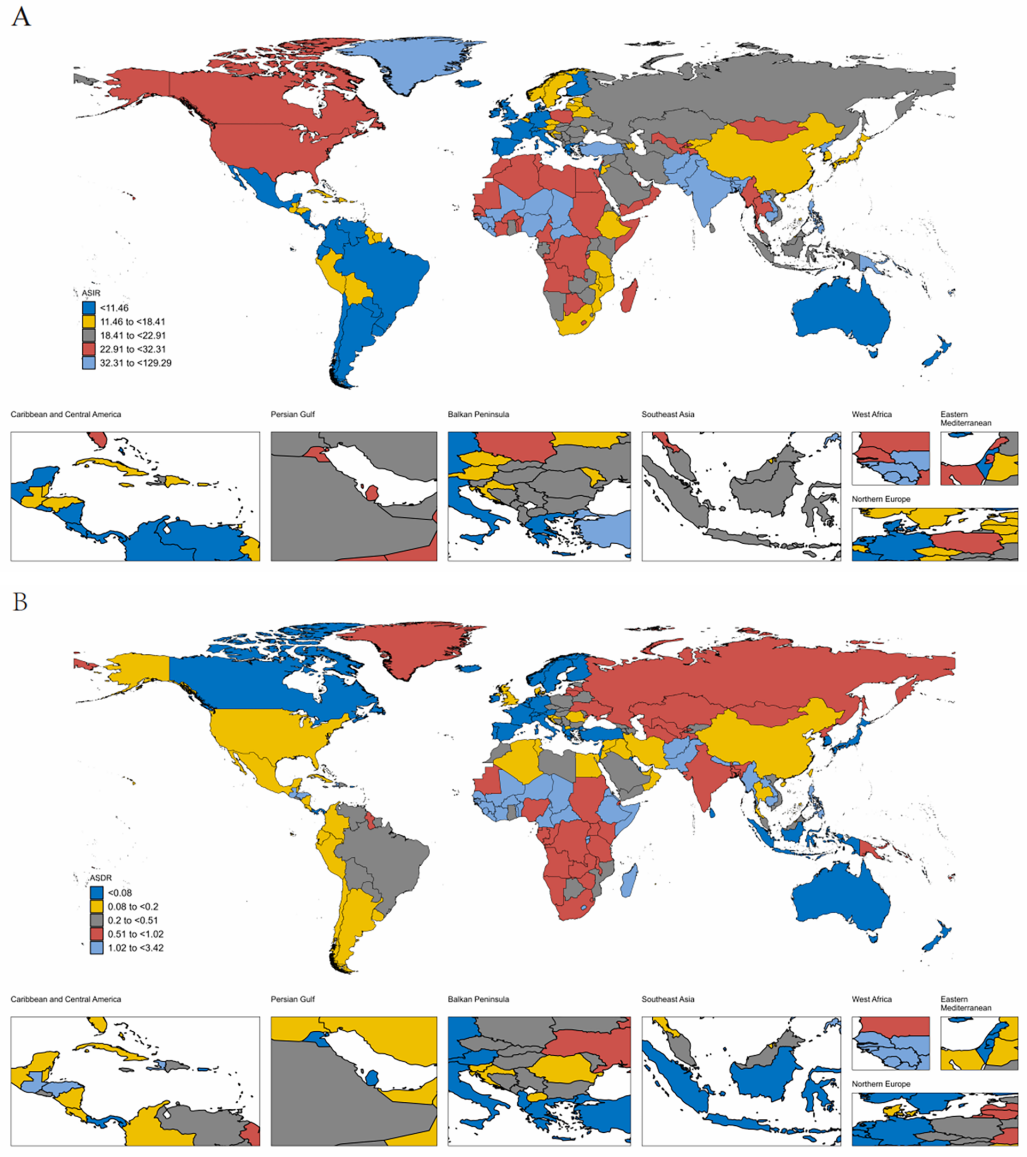
The ASIR **(A)** and ASDR **(B)** of PUD in women of reproductive age in 204 countries and territories in 2021. ASIR, age-standardized incidence rate; ASDR, age-standardized death rate.

At the national and regional level, the estimated ASIR and ASDR varied significantly across the 204 countries and territories, with Taiwan (Province of China) and Cambodia recording the highest ASIR and ASDR, and the lowest in Costa Rica and San Marino in 2021(Figure 2, Supplementary Tables S1–S2). From 1992 to 2021, the country with the greatest increase in ASIR for WCBA with PUD was Taiwan (Province of China) (4.02%) and greatest increase in ASDR was Thailand (7.43%) (Supplementary Table S3). Over the same period, El Salvador showed the most substantial decrease in ASIR for WCBA with PUD (−2.79%) and Qatar exhibit the most substantial decrease in ASDR (−10.23%). In addition, the pattern of changes in ASIR and ASDR for PUD among WCBA with increased SDI at the national level was similar to the pattern at the 21 regional levels (Figure 1B, Supplementary Figure S2B).

### Age-period-cohort effects on PUD morbidity and mortality in WCBA

**Figure 3:**
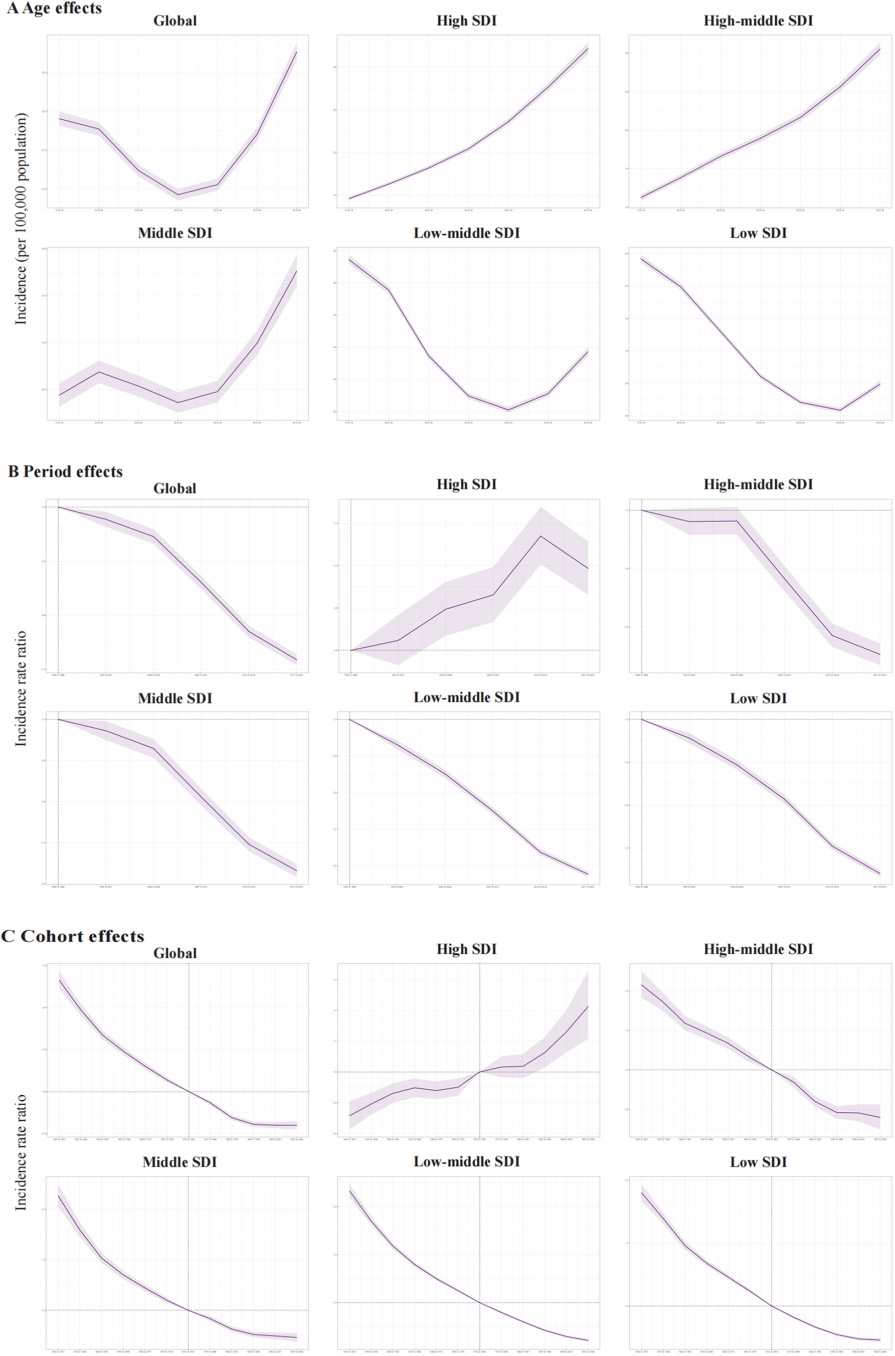
The age-period-cohort effects on the incidence of PUD in women of reproductive age in global and five SDI regions. **(A)** Longitudinal age curve; **(B)** period rate ratio; **(C)** cohort rate ratio.

**Figure 4:**
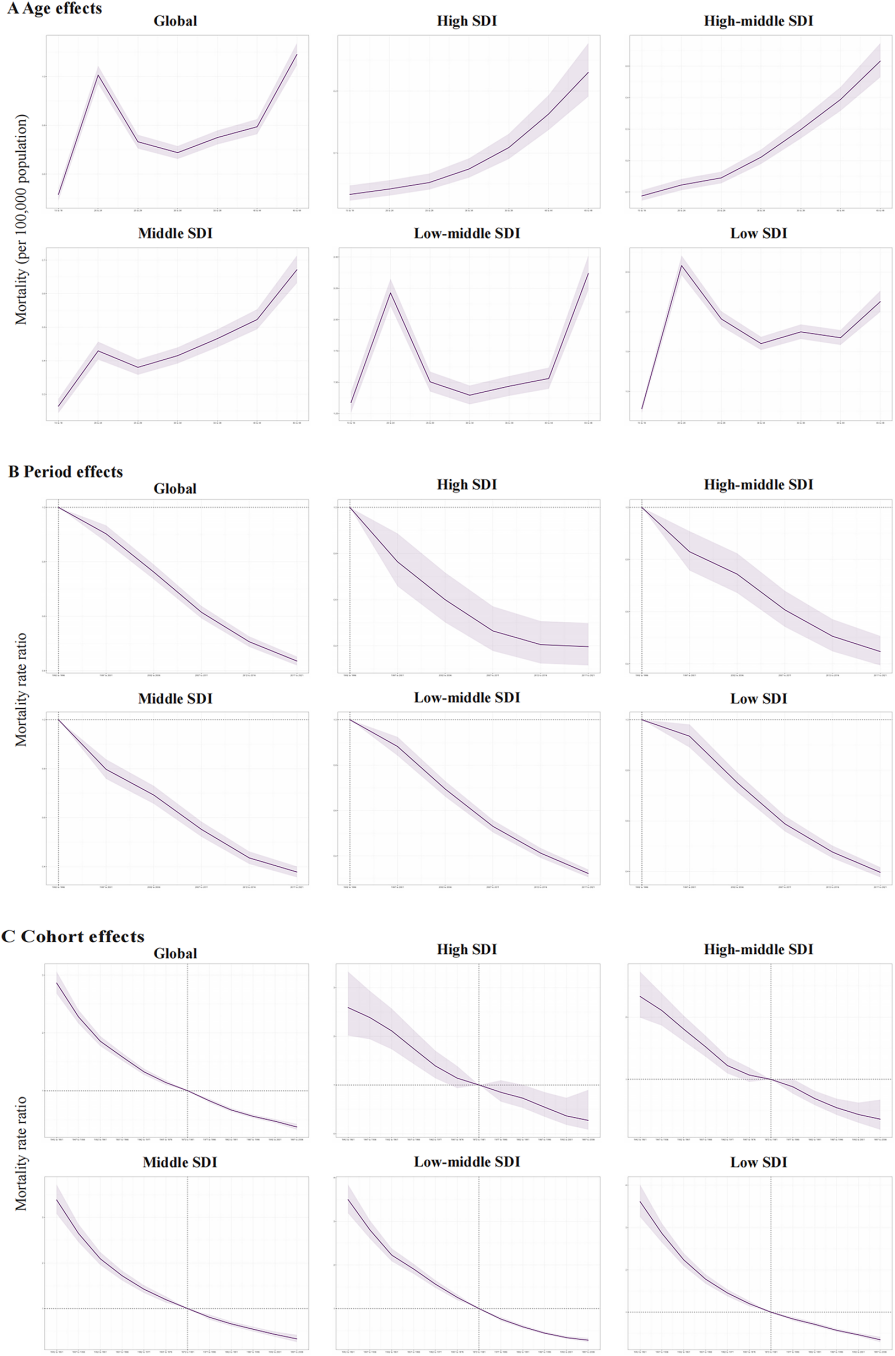
The age-period-cohort effects on the mortality of PUD in women of reproductive age in global and five SDI regions. **(A)** Longitudinal age curve; **(B)** period rate ratio; **(C)** cohort rate ratio.

The age, period, and birth cohort effects on PUD morbidity and mortality among WCBA derived from the APC model were demonstrated in Figures 3, 4. For the incidence risk of PUD in WCBA, similar patterns of age effects were observed across high and high-middle SDI quintiles, which is an increasing trend with age increase (Figure 3A). The incidence risk of middle SDI quintile exhibited scoop-shaped upward trend, while low-middle and low SDI quintiles exhibited scoop-shaped downward trend. The period effect indicated a declined in global morbidity risk since 1992 (Figure 3B). However, in comparison to other SDI regions, the high SDI regions exhibited a ladle-shaped upward trend, with the highest incidence rates observed during the 2012–2016 period and consistently higher incidence rates across all age groups. In the remaining four SDI regions, period effect has trended upward, indicating that the incidence of PUD has been effectively managed over time. The cohort effect showed a significant decrease in the successive birth cohort globally and across different SDI regions except in high SDI regions (Figure 3C). The cohort effect showed a significant increase in incidence risk from earlier birth cohorts to more recent birth cohorts in high SDI regions.

As for the mortality risk of PUD in WCBA, the risk increases with age like incidence risk, and reaches the highest in older people at 45–49 years (Figure 4A). The period effects presented a declining risk of mortality globally (Figure 4B). Regarding birth cohort effects, there was also a declining risk of mortality in successively birth cohorts globally (Figure 4C). Both of the period effects and cohort effects indicated mortality improvement.

### Frontier analysis of PUD in WCBA from 1992 to 2021

**Figure 5:**
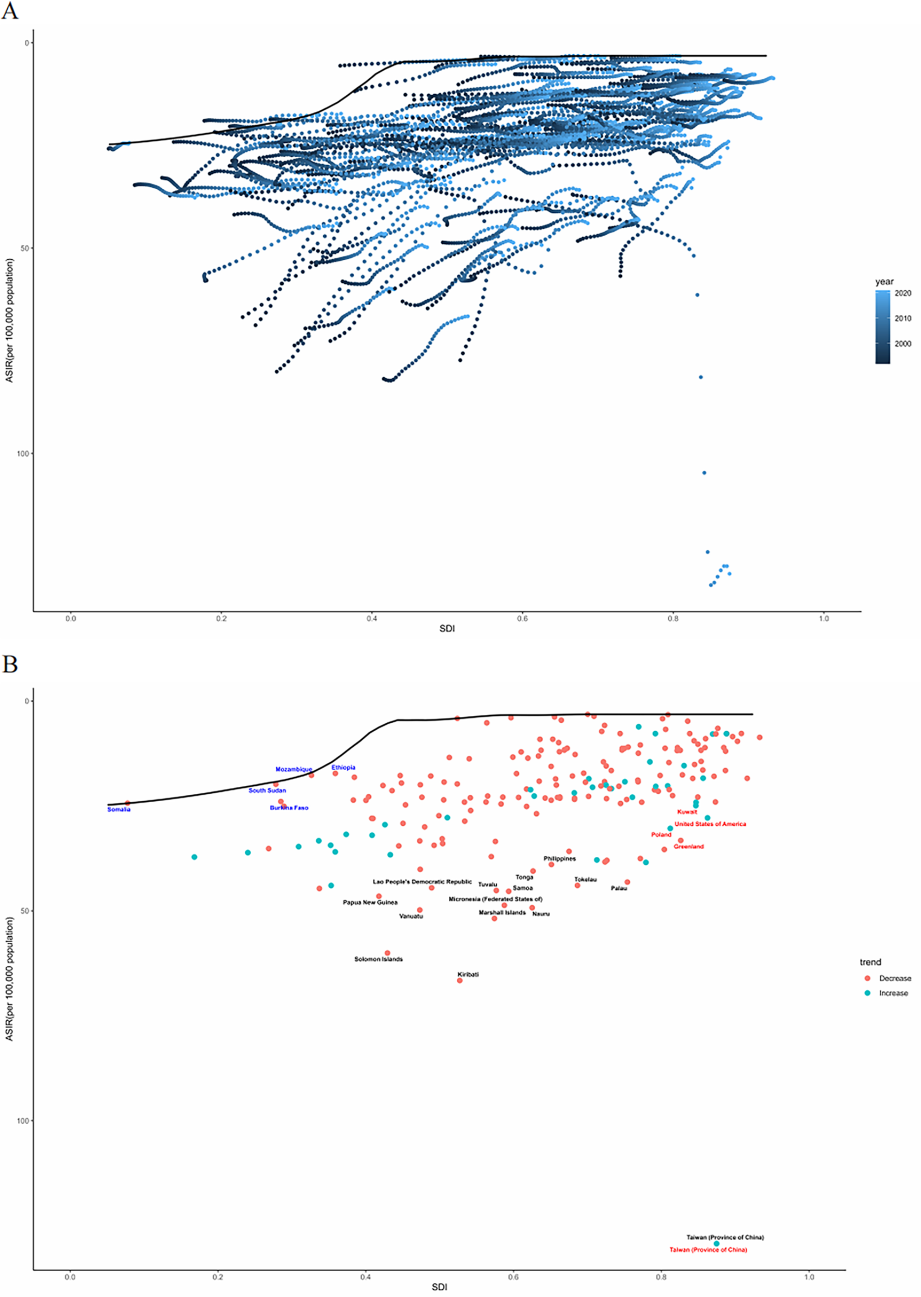
Frontier analysis based on the ASIR for PUD in women of reproductive age and SDI from 1992 to 2021 **(A)** and specifically in 2021 **(B)** ASIR, age-standardized incidence rate.

Figure 5A illustrates the unrealized health gains for countries or regions at varying levels of development from 1992 to 2021. Figure 5B depicts the burden and effective variance of ASIR in 2021 for countries across various levels of sociodemographic development. Effective differentials tend to increase with higher levels of sociodemographic development, indicating that countries or regions with varying sociodemographic development indices have greater potential for burden reduction.

### Predictions of PUD morbidity and mortality in WCBA for 2044

**Figure 6:**
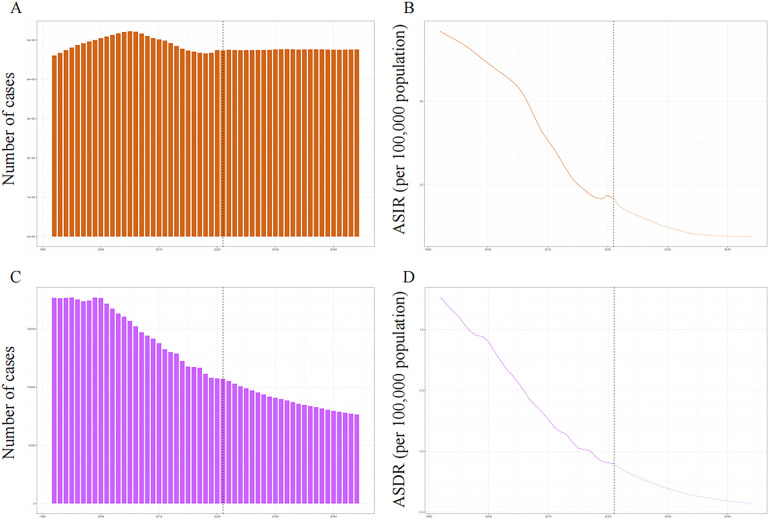
The global trends in incident number and ASIR **(A,B)**, deaths and ASDR **(C,D)** of PUD in women of reproductive age from 1992 to 2044. ASIR, age-standardized incidence rate; ASDR, age-standardized death rate.

The incidence number of PUD in WCBA were projected a slight increasing trend, while the number of death is on a clear downward trend (Figure 6). In 2044, the overall number of new PUD cases in WCBA should increase to 476,196. The ASIR and ASDR were expected to decline until 2044. In 2044, the ASIR should decrease to 21.90 and the ASDR should decrease to 0.17.

## Discussion

In this study, we calculated the ASIR and ASDR for PUD among WCBA from 1992 to 2021 using data from GBD 2021 and examined their trends at global, regional, and national levels. Given the intricate interplay between age-period-cohort effects, we analyzed the relative impact of these variables on trends in PUD morbidity and mortality. We employed frontier analyses to visualize the potential for each country or region to reduce the burden based on its level of development. Additionally, we projected global morbidity and mortality rates to 2044. These results of these studies are critical to rationalizing the allocation of healthcare resources.

In 2021, significant disparities were observed in PUD ASIR and ASDR across global, regional, and national levels for WCBA, indicating substantial regional variation likely attributable to factors such as ethnic differences, dietary variations, environmental exposures, and patterns of drug use (3). Notably, the clustering of PUD in relatively undeveloped areas is particularly interesting, where the ASIR of PUD in the WCBA shows a negative correlation with the SDI level. This finding is consistent with previous studies indicating that countries with lower levels of SDI may experience a greater burden of PUD (10). However, in the high-income North American regions, the ASIR was slightly elevated compared to that of high-middle SDI regions. Several potential reasons could explain this phenomenon. Firstly, WCBA in high SDI regions may experience various stressors, including life experiences and social learning, which disrupt bidirectional gut-brain axis signaling, resulting in dysregulated acid secretion, mucosal barrier impairment, and pathological progression (28). Similar to the prediction made by H. pylori positive, “mental vulnerability” has also been identified as a significant predictor of peptic ulcer development (29). Secondly, significant increasing trends have been observed in the prescription of NSAIDs within the US Ambulatory Care Setting (30, 31). Finally, PUD in WCBA are more likely to be diagnosed and treated due to the relatively universal health coverage in high SDI areas (32). Furthermore, the overall burden of PUD in WCBA may be significantly greater than what is currently observed, considering the limited data collection in regions with underdeveloped medical systems.

The global ASIR and ASDR for PUD among WCBA demonstrate a significant downward trend from 1992 to 2021, indicating that substantial progress has been achieved in the management of PUD over recent decades. At the regional level, the estimates of ASIR and ASDR for PUD among WCBA reveal varying trends across different regions. Notably, while the ASIR and ASDR showed declining trends from 1992 to 2021 in middle and low SDI regions, high SDI regions exhibited increasing incidence trends, suggesting ongoing issues in the etiology of PUD in WCBA in these areas. Factors such as diet and lifestyle, various stressors, the use of NSAIDs, changes in alcohol consumption and smoking patterns, rising obesity rates, and improvements in imaging techniques may collectively explain these differences (33). Furthermore, the estimates of ASIR and ASDR varied significantly among the 204 countries and territories, indicating considerable differences in the prevention and treatment of PUD across urban and border regions worldwide. Identifying the unique variations in trends within each country or region is essential for governments to enhance their healthcare systems in order to address the diverse healthcare needs associated with PUD among WCBA.

The age effect elucidates variations in incidence and mortality of PUD among the WCBA population, reflecting developments associated with growth and aging throughout the life course. Our study indicated that the risks of incidence and mortality gradually increase with advancing age; however, in the low-middle and low SDI regions, the risk of incidence decreases with age. Previous studies have documented similar patterns in the incidence and mortality rates associated with PUD. For instance, in Asia, the incidence of PUD has declined steadily over the past two decades across various ethnic groups, Chinese, and Indians (34). Other studies indicate that hospital admissions for complications of PUD disease have decreased in the 21st century, with an incidence rate of 79 cases per 100,000 people per year, compared to fewer than 30 cases per 100,000 people per year for these complications (35, 36). The reduction in complications related to peptic ulcer disease may be attributed to the extensive global utilization of anti-secretory drugs (32). The period effect indicates that both incidence and mortality rates have significantly decreased globally, with the exception of high SDI regions, where healthcare advancements may play a crucial role. Research on PUD has advanced significantly over the past few decades, both in terms of quantity and quality. Numerous clinical studies continue to advance the management of PUD (37, 38). Notably, the incidence risks for PUD in WCBA in high SDI regions have not been effectively managed over time. The cohort effects indicate that variations among different birth cohorts significantly influence the incidence and mortality of PUD among WCBA, reflecting alterations in exposure to environmental risk factors and lifestyle modifications associated with specific cohorts. In high SDI areas, the cohort effects on PUD incidence among WCBA demonstrated a decreasing trend from earlier to later birth cohorts. A potential reason for this trend is that later-born cohorts generally possess higher educational levels, encounter various stressors, and exhibit stronger disease awareness. Simultaneously, advancements in medical technology have contributed to a reduction in the risk of mortality associated with PUD. In summary, the overall decrease in PUD morbidity and mortality is encouraging for WCBA populations and their families globally. However, there is an urgent need for policymakers to prioritize healthcare resource allocation to reduce the rising PUD risk among WCBA populations in high SDI regions. Furthermore, it is essential to emphasize that WCBA in the later stages of their reproductive years should prioritize taking precautions against PUD.

We also employed frontier analysis to elucidate potential areas for improvement and disparities among countries based on their development status. Notably, low-SDI countries such as Somalia, Mozambique, the Democratic Republic of Congo, Niger, and Malawi have demonstrated exemplary management of the burden of these diseases through widespread use of proton-pump inhibitors and histamine2 receptor antagonists, and enhanced societal recognition of women's health needs has led to stronger research and public health initiatives for diseases affecting women (39). Despite resource constraints, these nations showcase effective strategies for improving health outcomes in challenging contexts. Effective strategies include widespread use of proton-pump inhibitors and histamine2 receptor antagonists, and enhanced societal recognition of women's health needs has led to stronger research and public health initiatives for diseases affecting women. Conversely, high-SDI countries, including Greenland, Poland, Latvia, Lithuania, and Estonia, have performed suboptimally in managing the burden of PUD relative to their developmental status. This situation underscores the urgent need to enhance optimization and reform in the formulation and implementation of health policy in these regions. From 2022 to 2044, our results indicate that the PUD ASIR and ASDR are expected to decrease in woman globally. However, the total number of incident cases is anticipated to rise, likely due to population growth NSAID overuse, and antibiotic resistance to H. pylori. Therefore, governments should consider the potential health impacts of growing economically developed populations and demographic changes when formulating or revising health prevention strategies.

This study possesses several strengths. First, it encompasses a wide geographic area and a substantial time span, drawing from an extensive datasets. Second, the estimation of age-standardized morbidity and mortality rates of PUD in WCBA addresses the heterogeneity of age structures, thereby facilitating valid comparisons across different regions. Third, the application of age-time-cohort modeling allows for the distinction of the independent effects of age, period, and cohort on the incidence and mortality risks associated with PUD in WCBA.

However, this study is not without limitations. Firstly, deficiencies in the healthcare systems of less developed countries may result in a misjudgment of the disease burden. Secondly, our reliance on modeling procedures for estimations means that the models and parameter settings selected may have influenced the results. Thirdly, the lack of detailed data prevented exploration of epidemiological trends in PUD in WCBA at the subnational level. Fourthly, some symptoms of PUD are mild and self-limiting, which may lead to unrecognized cases among individuals who do not seek medical care, thereby underestimating the overall burden of PUD. Fifthly, like other APC analyses, this study is subject to ecological fallacy, interpretations of population-level results do not necessarily translate to individual cases. Finally, since data were extrapolated from existing epidemiological sources, caution is warranted in interpreting these results in real-world contexts.

## Conclusion

In summary, our analysis highlights significant global disparities in PUD trends among WCBA (1992–2021), with high-income countries exhibiting an increased trend of ASIR and developing countries facing poor disease management over the past three decades. Additionally, we forecast an increase in the number of incident and death cases of PUD in WCBA by 2044. Addressing these disparities requires tailored interventions: high-income regions should prioritize antibiotic stewardship and NSAID prescribing guidelines, while low-income regions must implement community-based H. pylori screening into maternal health programs. Future efforts should consider gender-specific social determinants, and promote equity-focused innovations in diagnostics and treatment.

## Data Availability

The original contributions presented in the study are included in the article/Supplementary Material, further inquiries can be directed to the corresponding authors.
